# Time interval of esomeprazole and dual antiplatelet therapy in patients with cardiocerebrovascular diseases

**DOI:** 10.1097/MD.0000000000037205

**Published:** 2024-03-01

**Authors:** Hyunsoo Kim, Moon-Hwa Park, Joon-Tae Kim

**Affiliations:** a Department of Neurology, Chonnam National University Hospital, Gwangju, Korea; b Hanmi Pharmaceutical Co., Ltd., Seoul, Korea.

**Keywords:** administration interval, dual antiplatelet therapy, esomeprazole

## Abstract

Dual antiplatelet therapy (DAPT) with the combination of clopidogrel and aspirin is recommended for preventing secondary ischemic events in patients with acute coronary syndrome (ACS) or acute ischemic stroke (AIS). Proton pump inhibitors (PPIs) are suggested as preventive treatment for these patients. Due to clopidogrel-PPI interactions, separating their administration might be considered. However, a paucity of studies has been conducted to investigate the outcome differences between concurrent and interval-based use in ACS and AIS patients. Our study aimed to evaluate clinical outcomes based on administration timing. This study included patients with ACS or AIS onset or recurrence of within the last month. Patients who were expected to receive DAPT for at least 6 months and who were currently taking or planning to take esomeprazole were included. Patients were divided into Group 1 (interval administration group, IA group) and Group 2 (concurrent administration group, CA group) according to the interval between esomeprazole and DAPT administration. The time interval was based on 12 hours. The primary outcome was the occurrence of major adverse cardiocerebrovascular events (MACCEs), and safety outcomes were defined as major bleeding, minor bleeding and gastrointestinal bleeding and intracranial hemorrhage. A total of 3600 patients completed this study. The proportions of patients in the 2 groups were as follows: CA group, 99% (n = 3489) and IA group, 1% (n = 111). The primary outcome occurred in 0.9% of patients in the IA group and 1.8% of patients in the CA group (*P* = .51). There was no significant distinction in the overall bleeding risk of the CA group compared to that of the IA group (2.75% in the CA group and 2.70% in the IA group). Additionally, there was no significant difference observed between the 2 groups for safety outcomes. This multicenter, prospective, observational study that enrolled patients with ACS or AIS demonstrated that there was no significant difference in the occurrence of MACCEs and bleeding issues within 6 months according to the medication administration interval. The majority of patients with DAPT were taking PPIs simultaneously in real-world practice.

## 1. Introduction

Dual antiplatelet therapy (DAPT) with the combination of clopidogrel and aspirin is recommended for preventing secondary ischemic events in patients with acute coronary syndrome (ACS) or acute ischemic stroke (AIS).^[1,2]^ However, long-term use of DAPT may increase the risk of bleeding.^[3,4]^ Therefore, for patients receiving DAPT, it is recommended that preventive treatment with proton pump inhibitors (PPIs) be considered for those at high risk of gastrointestinal injury.^[5]^ However, there are concerns about drug interactions between PPIs and clopidogrel, which is activated by CYP2C19, an enzyme involved in the metabolism of PPIs and clopidogrel.^[6–8]^ When used together, PPIs can decrease the activation of clopidogrel by inhibiting the process of inactivation, which may reduce its antiplatelet effect and even decrease its clinical usefulness.^[9]^ Although the results from clinical trials are inconsistent,^[10–13]^ this is still a concern.

It is theoretically possible to minimize potential drug interactions by spacing out the administration of PPIs and clopidogrel by 12–20 hours, as they have a short duration of existence in the blood, which may prevent the competitive inhibition of CYP metabolism.^[14–16]^ In a study where esomeprazole was administered in the morning and clopidogrel was administered at night, there was no difference in the incidence of ischemic events compared to the administration of clopidogrel alone.^[14]^ However, a paucity of studies has been conducted to investigate the difference in clinical outcomes between the concurrent use and interval-based use of PPIs and clopidogrel in patients with ACS or AIS treated with clopidogrel-aspirin DAPT.

This study therefore aimed to evaluate and compare the incidence of major adverse cardiocerebrovascular events (MACCEs), major bleeding, and other clinical outcomes based on the administration schedule (12-hour intervals or simultaneous administration) of esomeprazole for 6 months in patients with ACS or AIS receiving DAPT.

## 2. Methods

### 2.1. Study design

This was a multicenter, prospective, observational study to evaluate the incidence of MACCEs according to the time interval of medication administration in patients with ACS or AIS who received DAPT for secondary prevention and esomeprazole for the prevention of gastrointestinal bleeding. From April 2019 to June 2022, this study was conducted in 75 centers in South Korea (study number: HM-ESO-OS-01).

### 2.2. Participants

Patients who have visited the hospital due to ACS or AIS onset or recurrence within the last month before enrollment, were expected to receive DAPT (clopidogrel + aspirin) for at least 6 months and were receiving or scheduled to receive 20 mg esomeprazole as a concomitant treatment for the prevention of gastrointestinal bleeding were eligible for this study. More detailed inclusion and exclusion criteria are presented in the Supplemental Methods.

The patients were divided into 2 groups according to the interval between the administration of esomeprazole and DAPT: Group 1 (interval administration group, IA group): patients for whom esomeprazole was administered before breakfast and clopidogrel was administered after dinner and those for whom the interval between clopidogrel and esomeprazole administration was ≥ 12 hours; and Group 2 (concurrent administration group, CA group): patients administered clopidogrel and esomeprazole simultaneously or taking clopidogrel-esomeprazole before-after meals (intervals < 12 hours).

**Figure 1.**
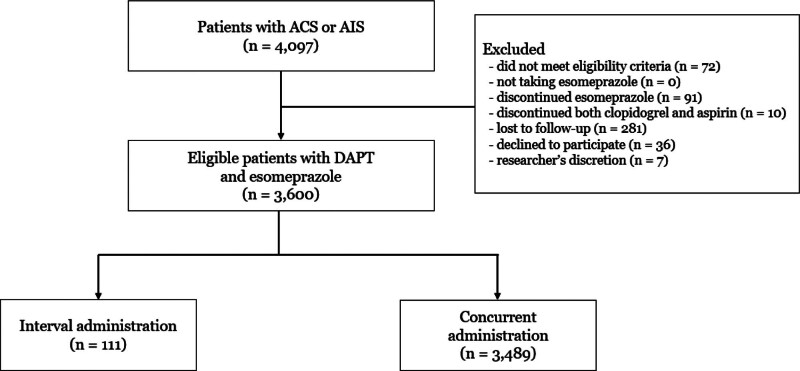
Flow diagram of the study population.

Among the criteria proposed by the Academic Research Consortium for High Bleeding Risk group, which predicts bleeding risk after percutaneous coronary intervention, we defined a group showing one or more major criteria as a high-risk group.^[17]^ We defined high bleeding risk in patients to individuals meeting one or more of the followings: Age over 75 years, a documented history of cerebrovascular accident, presence of chronic kidney disease or end-stage renal disease, presence of malignancy, presence of anemia or thrombocytopenia, current use of antiplatelet or anticoagulant medications. A detailed patient selection flowchart is shown in Figure 1.

### 2.3. Ethics and data availability statement

This study was approved by the Institutional Review Board (IRB) of all participating hospitals, including Chonnam National University Hospital. Written informed consent was obtained from all the participants or their caregivers.

### 2.4. Data collection

Demographic, clinical, and laboratory data were prospectively collected by dedicated research nurses or physicians. The following vascular risk factors were identified: age, sex, hypertension, diabetes mellitus (DM), dyslipidemia, current smoking, current drinking, a previous history of stroke or transient ischemic attack, coronary artery diseases, gastrointestinal diseases, and medication history.

### 2.5. Outcome

The primary outcome was the occurrence of MACCEs, a composite outcome of stroke (either ischemic or hemorrhagic), myocardial infarction (MI), vascular death, repeat cardiac revascularization procedures, and hospitalization due to other cardiovascular events up to 6 months after enrollment. Secondary outcomes were the occurrence of the following individual outcomes within 6 months after enrollment: vascular death, MI, stroke (either ischemic or hemorrhagic), repeat cardiac revascularization procedures, and hospitalization due to other cardiovascular events.

The safety outcomes were the occurrence of major bleeding, intracranial hemorrhage, gastrointestinal (GI) bleeding, and minor bleeding. Major bleeding was defined as follows: intracranial hemorrhage, bleeding requiring blood transfusion, a decrease of ≥ 5 g/dL in the hemoglobin level or a decrease of ≥ 15% in the hematocrit level. GI bleeding was defined as overt hematemesis or melena/hematochezia and occult bleeding by stool examination. We used a survey to confirm overall medication adherence and adherence to the prescribed dosage regimen for treatment medication.

### 2.6. Sample size estimation

The estimation of event rates in the treatment groups was based on findings from the DAPT + PPI group vs the DAPT-only group in the results of the meta-analysis by Hu et al (9.4% vs 7.7%, respectively). We calculated the required number of study participants using a 95% confidence interval for a 2:1 allocation ratio between the 2 groups and a 4% length of both sides of the confidence interval. The required number of participants for this study was 3275. Taking into account an estimated dropout rate of approximately 15%, a total of 4002 trial participants (2668 in the CA group and 1334 in the IA group) were recruited for the study.

### 2.7. Statistical analysis

Baseline characteristics and outcomes were compared between the treatment groups (CA group vs IA group) by using the chi-square test, ANOVA, or Kruskal–Wallis test according to the type of variable. The event probability of vascular outcomes within 6 months after medication use was calculated by using the Kaplan–Meier method, and the log-rank test was performed to analyze differences between the groups. Hazard ratios (HRs) and 95% confidence intervals (95% CIs) for vascular outcomes were analyzed using the Cox proportional hazards model. Adjustments were made for the following predetermined variables with clinically relevant associations with the outcome variables: age, sex, BMI and the index event.

We conducted inverse probability of treatment weighting (IPTW) to stabilize differences between the 2 groups. Subsequently, based on these results, we compared the outcomes of the 2 groups using cox proportional hazard regression model. Stabilized IPTW was utilized, and to mitigate the issue of escalating type 1 error, a stabilized version with unbiased estimates was incorporated into the cox regression model. The selection of variables for propensity score matching (PSM) was guided by the standardized mean difference. Variables deemed suitable for PSM were those with a *P* value of .1 or less. The decision to exclude some variables from PSM correction was based on the inapplicability of the results from the cox proportional hazard model when most variables in Table 1 were included.

**Table 1:** General characteristics of subjects according to the index events.

	Total	Interval-based use	Concurrent use	*P* ^*^
N	3600	111	3489	
Age, mean (SD)	65.46 (11.65)	65.77 (13.41)	65.45 (11.59)	.81
Male, n (%)	2585 (71.81)	77 (69.37)	2508 (71.88)	.56
Body weight, mean (SD)	66.74 (11.81)	66.95 (12.29)	66.74 (11.80)	.89
Height, mean (SD)	164.02 (8.74)	164.20 (8.54)	164.02 (8.75)	.87
BMI, mean (SD)	24.70 (3.36)	24.68 (3.25)	24.70 (3.36)	.96
Medical history, n (%)				
Smoking	904 (27.08)	16 (22.22)	888 (27.19)	.35
Alcohol	1048 (32.36)	23 (32.86)	1025 (32.34)	.93
H. pylori infection	10 (1.01)	0 (0.00)	10 (1.05)	1.00
CAD (STEMI/NSTEMI/UA)	263 (7.31)	10 (9.01)	253 (7.25)	.48
Stroke (ischemic or hemorrhagic)	98 (2.72)	4 (3.60)	94 (2.69)	.54
Index events				
STEMI	386 (10.73)	11 (9.91)	375 (10.75)	.78
NSTEMI	568 (15.78)	25 (22.52)	543 (15.57)	.05*
UA	1548 (43.01)	48 (43.24)	1500 (43.00)	.96
Ischemic stroke	1097 (30.48)	27 (24.32)	1070 (30.68)	.15
Concurrent diseases				
STEMI	435 (12.08)	12 (10.81)	423 (12.12)	.68
NSTEMI	588 (16.33)	25 (22.52)	563 (16.14)	.07
UA	1592 (44.22)	51 (45.95)	1541 (44.17)	.71
Ischemic stroke	1176 (32.67)	28 (25.23)	1148 (32.90)	.09
Hemorrhagic stroke	14 (0.39)	0 (0.00)	14 (0.40)	1.00
Prior medications				
Statin	690 (19.17)	16 (14.41)	674 (19.32)	.20
NSAIDS	68 (1.89)	2 (1.80)	66 (1.89)	1.00
High bleeding risk				.52
No, n (%)	2373 (67.9)	71 (65.1)	2302 (68.1)	
1 or more, n (%)	1119 (32.0)	38 (34.9)	1081 (32.0)	

Values are the means and standard deviations.

CAD = coronary artery disease, MI = myocardial infarction, NSTEMI = non-ST elevation myocardial infarction, STEMI = ST elevation myocardial infarction, UA = unstable angina.

Statistical analyses were performed with SAS^®^ Enterprise Guide (8.3 version), 9.4 64-bit (SAS Institute Inc., Cary, NC).

## 3. Results

### 3.1. General characteristics

Of the 4097 patients with ACS or AIS who provided informed consent, 3600 (mean age, 65.5 ± 11.7 years; male, 71.8%) completed this study. The proportions of patients in the 2 groups were as follows: CA group, 99% (n = 3489) and IA group, 1% (n = 111). The demographic and clinical characteristics of the subjects according to the interval between esomeprazole and DAPT administration are shown in Table 1. Both groups were not significantly different regarding general characteristics except for the index event of non-STEMI.

### 3.2. Outcomes

The mean follow-up (FU) period for all patients was 191.0 days (±38.7 days), and 99.8% of patients completed the FU. Overall, MACCEs within 6 months were observed in 61 patients (1.75%) from the entire patient population. Among these events, CV mortality occurred in 2 patients (0.06%), MI occurred in 4 patients (0.11%), stroke occurred in 16 patients (0.46%), repeated revascularization was performed in 6 patients (0.17%), and hospitalization was needed for 34 patients (0.97%). Bleeding occurred in 84 patients (2.41%), with major bleeding occurring in 12 patients (0.3%), minor bleeding occurring in 71 patients (2.0%), and GI bleeding occurring in 16 patients (0.5%).

**Figure 2.**
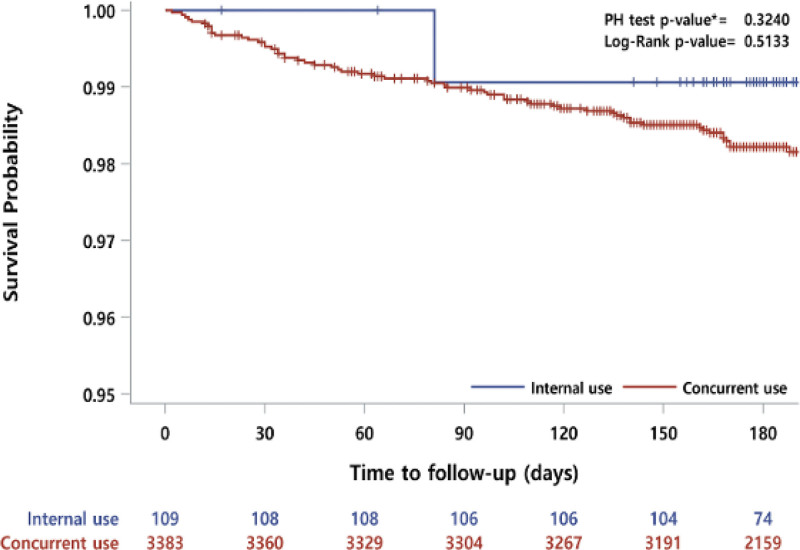
Kaplan–Meier curves for primary outcome within 6 months according to the administration method.

The outcomes of each treatment group are presented in Table 2. The primary outcome occurred in 0.9% of patients in the IA group and 1.8% of patients in the CA group (*P* = .51). The Kaplan–Meier curves for these groups are shown in Figure 2. There was no significant difference observed between the 2 groups. Regarding safety outcomes, there was no major bleeding in the IA group, and 0.35% of the patients in the CA group experienced major bleeding. Minor bleeding occurred in 2.75% of the patients in the IA group and 2.01% of the patients in the CA group. GI bleeding did not occur in the IA group, while it occurred in 0.47% of the patients in the CA group (*P* = .48). In addition, high bleeding risk event did not occur in IA group, while it occurred in 0.19% of the patients in the CA group (*P* = .79) (Table 2).

**Table 2:** Event rates within 6 mo after observation.

	All patients	Interval use	Concurrent use	
	Event rate, n (%, 95% CI)	Event rate, n (%, 95% CI)	Event rate, n (%, 95% CI)	*P* ^*^
Primary efficacy outcome				
MACCE	61 (1.75) [1.34, 2.24]	1 (0.92) [0.02, 5.01]	60 (1.77) [1.36, 2.28]	.5133
Secondary outcome				
Stroke	16 (0.46) [0.26, 0.74]	0 [0.00, 3.33]	16 (0.47) [0.27, 0.77]	.4766
MI	4 (0.11) [0.03, 0.29]	0 [0.00, 3.33]	4 (0.12) [0.03, 0.30]	.7146
Vascular death	2 (0.06) [0.01, 0.21]	0 [0.00, 3.33]	2 (0.06) [0.01, 0.21]	.7999
Primary safety outcome				
Major bleeding	12 (0.34) [0.18, 0.60]	0 [0.00, 3.33]	12 (0.35) [0.18, 0.62]	.5466
Secondary safety outcome				
Minor bleeding	71 (2.03) [1.59, 2.56]	3 (2.75) [0.57, 7.83]	68 (2.01) [1.56, 2.54]	.5709
GI bleeding	16 (0.46) [0.26, 0.74]	0 [0.00, 3.33]	16 (0.47) [0.27, 0.77]	.4775

GI = gastrointestinal, MACCEs = major adverse cardiac and cerebrovascular events, MI = myocardial infarction.

Compared with the IA group, the CA group was not significantly associated with the risk of MACCEs within 6 months in unadjusted and adjusted analyses (HR 1.91 [0.26–13.79], adjusted HR 1.23 [0.15–10.01]) (Table 3). In comparison to the IA group, the CA group did not have a significantly reduced risk of minor bleeding (unadjusted HR 0.72 [0.23–2.28]; adjusted HR 0.73 [0.20–2.65]).

**Table 3:** Cox proportional hazard regression analysis.

	Unadjusted HR (95% CI)	P	Adjusted HR (95% CI) Model 1	P	Adjusted HR (95% CI) Model 2	*P*
MACCEs						
Interval-based use	Ref		Ref		Ref	
Concurrent use	1.91 [0.26, 13.79]	0.5207	1.23 [0.15, 10.01]	0.8463	1.16 [0.14, 9.36]	.8899
Stroke						
Interval-based use	Ref		Ref		Ref	
Concurrent use	NA	NA	NA	NA	NA	NA
MI						
Interval-based use	Ref		Ref		Ref	
Concurrent use	NA	NA	NA	NA	NA	NA
Vascular death						
Interval-based use	Ref		Ref		Ref	
Concurrent use	NA	NA	NA	NA	NA	NA
Major bleeding						
Interval-based use	Ref		Ref		Ref	
Concurrent use	NA	NA	NA	NA	NA	NA
Minor bleeding						
Interval-based use	Ref		Ref		Ref	
Concurrent use	0.72 [0.23, 2.28]	0.5727	0.73 [0.20, 2.65]	0.6291	0.70 [0.19, 2.56]	.5921
Minor bleeding						
Interval-based use	Ref		Ref		Ref	
Concurrent use	NA	NA	NA	NA	NA	NA

MACCEs = major adverse cardiac and cerebrovascular events, MI = myocardial infarction.

IPTW analysis confirmed that the standardized mean difference approached 0, indicating a relatively balanced matching of the 2 groups (Supplemental figure 2, http://links.lww.com/MD/L630). The analysis using IPTW revealed a primary outcome of 0.9% in the IA group and 1.8% in the CA group (*P* = .51, Supplemental table 1, http://links.lww.com/MD/L624). Following the application of PSM or IPTW, there were no statistically significant differences observed in both the primary outcome and secondary outcomes, which encompassed the occurrence of stroke, MI, and vascular death (Supplemental table 2, http://links.lww.com/MD/L625). The cox proportional hazard regression model and Kaplan–Meier curves for these groups demonstrated no significant difference between the 2 (Supplemental table 3, http://links.lww.com/MD/L626 and Supplemental Figure 1, http://links.lww.com/MD/L628). These findings, detailed in the supplemental materials, closely align with the results of the main outcome analysis.

## 4. Discussion

Our study, a multicenter, prospective, observational study that enrolled patients with coronary artery diseases or stroke, demonstrated that there was no significant difference in the occurrence of MACCEs or major bleeding within 6 months when comparing the simultaneous use of esomeprazole and DAPT with the interval-based use of the 2 medications. There was no discernible distinction in the overall bleeding risk of the CA group compared to the IA group (2.75% of patients in the CA group and 2.70% of patients in the IA group). Additionally, we found that the majority of the 99% of patients who received DAPT and esomeprazole were taking esomeprazole and DAPT simultaneously. These findings reflect the real-world situation of the use of esomeprazole with DAPT and suggest that this practice may not be clinically significant in terms of risks and benefits.

The use of esomeprazole with clopidogrel has been a topic of debate due to the potential for drug interactions and reduced effectiveness of clopidogrel, which may lead to an increased risk of cardiovascular events. There have been concerns regarding the interaction between PPIs and clopidogrel in patients with ACS or AIS, as it is associated with CYP enzymes and may potentially reduce the effectiveness of clopidogrel.^[18]^ In some studies, omeprazole was found to decrease the effectiveness of clopidogrel and increase the risk of vascular events.^[19]^ However, pantoprazole and esomeprazole do not have any attenuating effects on the platelet response to clopidogrel.^[10]^ Subsequent randomized controlled trials investigating the relationship between the use of PPIs and clopidogrel have shown that PPI use significantly reduces the incidence of GI bleeding without affecting the clinical efficacy of clopidogrel.^[13]^ In our study, when comparing the IA group to the CA group, there was no significant difference in GI bleeding or minor bleeding. However, there appeared to be an association between a reduction of approximately 20% in the point estimate of MACCEs. There was a difference of 0.85% in the incidence rate of MACCEs within 6 months between the IA group and the CA group.

The interval-based administration of PPIs and clopidogrel, with a theoretical interval of 12–20 hours, can help prevent competitive inhibition of CYP metabolism and minimize potential drug interactions due to their short duration of existence in the bloodstream.^[14]^ In a previous study, varying the dose and timing of esomeprazole administration could provide appropriate acid inhibition for the symptom pattern of individual patients with gastroesophageal reflux disease.^[20]^ Our study is valuable because it demonstrated, in real clinical practice, that there was no significant difference in the clopidogrel response when comparing the IA group to the CA group. Additionally, our study is noteworthy because it specifically selected patients receiving clopidogrel plus aspirin to investigate the effects of interval-based administration on bleeding. In a recent single-center study, for coronary heart disease patients on clopidogrel therapy for at least 1 year, the intermittent use of PPIs was associated with a lower risk of stroke, major adverse cardiovascular events (MACE), and net adverse clinical events compared to patients with sustained PPI use.^[21]^ However, our study differed from that study, as it was not retrospective in nature and specifically compared the concomitant administration and interval-based administration of esomeprazole and DAPT rather than comparing sustained vs intermittent PPI use.

Furthermore, our study found that 99.5% of the total participants received esomeprazole and clopidogrel concurrently. In real practice, interval-based administration (esomeprazole in the morning and clopidogrel in the evening) was observed in <1% of the population, indicating that clinicians prioritize compliance and adherence over drug interactions in patients with vascular diseases. Compliance is considered important when considering efficacy. Although the interval-based administration of PPIs and clopidogrel appears to reduce their interaction, our findings suggest that in real-world clinical practice, the convenience of drug administration may be more important.

There are several limitations in our study. First, our research was underpowered due to the limited sample size of the IA group. Second, our dataset does not include platelet function test data for individual patients, raising the possibility that this factor may have impacted influenced the results. Third, although this study was prospectively collected, it was an observational study design, so it was not able to control the various confounding factors that affected the results. Additionally, our study was restricted to Korea, resulting in limitations regarding race and ethnic generalizability. Another limitation is that outcome measurements were not blinded. It seems necessary to conduct further studies comparing the efficacy and stability of the 2 groups in the future.

In conclusion, our study demonstrated that in real-world clinical practice, the concomitant use of PPIs and clopidogrel (plus aspirin) is overwhelmingly more prevalent than interval-based use in ACS and AIS patients. Although our study was underpowered, no significant differences were observed in terms of efficacy and safety between the 2 treatment groups of patients with ACS and AIS. However, to confirm these findings, larger-scale studies will be necessary in the future.

## Author contributions

**Conceptualization:** Joon-Tae Kim.

**Data curation:** Moon-Hwa Park.

**Formal analysis:** Moon-Hwa Park.

**Methodology:** Moon-Hwa Park, Joon-Tae Kim.

**Supervision:** Joon-Tae Kim.

**Writing – original draft:** Hyunsoo Kim, Joon-Tae Kim.

**Writing – review & editing:** Hyunsoo Kim, Joon-Tae Kim.

## Supplementary Material










