# Supplementary material for: Time interval of esomeprazole and dual antiplatelet therapy in patients with cardiocerebrovascular diseases
Source: Medicine (Baltimore). 2024 Mar 1;103(9):e37205. doi: 10.1097/MD.0000000000037205 (PMC10906606; doi:10.1097/MD.0000000000037205)
Supplement: Supplementary file 1 [file medi-103-e37205-s001.docx]

Supplementary Figure 2. Assessment of Balance after propensity score matching via standardized mortality ratio weight method.

## IPTW (Stabilized)


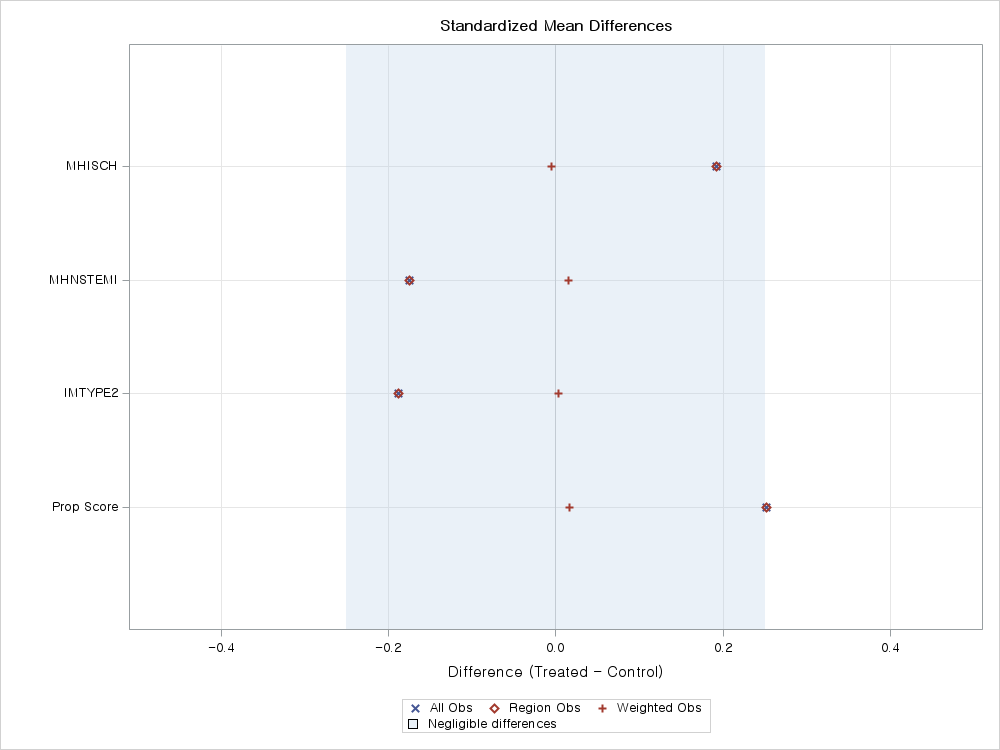


IPTW = inverse probability treatment weight
