# Supplementary material for: Time interval of esomeprazole and dual antiplatelet therapy in patients with cardiocerebrovascular diseases
Source: Medicine (Baltimore). 2024 Mar 1;103(9):e37205. doi: 10.1097/MD.0000000000037205 (PMC10906606; doi:10.1097/MD.0000000000037205)
Supplement: Supplementary file 2 [file medi-103-e37205-s002.docx]

Supplemental table 1. Inclusion and exclusion criteria

1. Inclusion and exclusion criteria

1. Inclusion criteria
2. Adults aged 19 years and older
3. Patients^*^ who visited a hospital within the past month due to the onset or recurrence of acute coronary syndrome or cerebral infarction and were expected to receive antiplatelet therapy (aspirin + clopidogrel) for a minimum of 6 months. These patients were currently receiving or scheduled to receive combination therapy with 20 mg esomeprazole for the prevention of gastrointestinal bleeding.

^*^ These criteria applied to patients who had started or were scheduled to receive the following treatment within the month prior to study enrollment.

1) Patients who were receiving dual antiplatelet treatment (DAPT) with aspirin + clopidogrel

2) Patients who were receiving DAPT and PPI combination therapy.

However, for patients in categories 1) and 2), at the time of registration, they must have been receiving DAPT with aspirin + clopidogrel to treat acute coronary syndrome or cerebral infarction, along with concurrent treatment with 20 mg esomeprazole.

3) Patients who were scheduled to receive DAPT and concurrent treatment with 20 mg esomeprazole.

1. Patients who provided informed consent to participate in the study.
2. Exclusion criteria
3. Patients with active bleeding or bleeding diatheses (peptic ulcer disease, intracranial hemorrhage, hemophilia, gastrointestinal bleeding, urogenital bleeding, hemoptysis, vitreous bleeding).
4. Patients with severe hepatic impairment.
5. Patients with severe renal impairment.
6. Patients with severe heart failure.
7. Patients who were unable to take or had contraindications to aspirin, clopidogrel, or esomeprazole due to allergic reaction or galactose intolerance.
8. Patients in the third trimester of pregnancy or those who were lactating.
