# Supplementary material for: Time interval of esomeprazole and dual antiplatelet therapy in patients with cardiocerebrovascular diseases
Source: Medicine (Baltimore). 2024 Mar 1;103(9):e37205. doi: 10.1097/MD.0000000000037205 (PMC10906606; doi:10.1097/MD.0000000000037205)
Supplement: Supplementary file 3 [file medi-103-e37205-s003.docx]

**Supplementary Table 2. Event rates within 6 months after observation**

## 1:1 matching PSM

|  | All patients | Interval-based use | Concurrent use |  |
| --- | --- | --- | --- | --- |
|  | Event rate,  n (%, 95% CI) | Event rate,  n (%, 95% CI) | Event rate,  n (%, 95% CI) | P^*^ |
| Primary efficacy outcome |  |  |  |  |
| MACCEs | 2 (0.92)  [0.11, 3.27] | 1 (0.92)  [0.02, 5.01] | 1 (0.92)  [0.02, 5.01] | 0.9921 |
| Secondary outcome |  |  |  |  |
| Stroke | 0  [0.00, 1.68] | 0  [0.00, 3.33] | 0  [0.00, 3.33] | NA |
| MI | 1 (0.46)  [0.01, 2.53] | 0  [0.00, 3.33] | 1 (0.92)  [0.02, 5.01] | 0.3173 |
| Vascular death | 0  [0.00,1.68] | 0  [0.00, 3.33] | 0  [0.00, 3.33] | NA |

## IPTW(stabilized)

|  | All patients | Interval-based use | Concurrent use |  |
| --- | --- | --- | --- | --- |
|  | Event rate,  n (%, 95% CI) | Event rate,  n (%, 95% CI) | Event rate,  n (%, 95% CI) | P^*^ |
| Primary efficacy outcome |  |  |  |  |
| MACCEs | 61 (1.75)  [1.34, 2.24] | 1 (0.92)  [0.02, 5.01] | 60 (1.77)  [1.36, 2.28] | 0.5133 |
| Secondary outcome |  |  |  |  |
| Stroke | 16 (0.46)  [0.26, 0.74] | 0  [0.00, 3.33] | 16 (0.47)  [0.27, 0.77] | 0.4898 |
| MI | 4 (0.11)  [0.03, 0.29] | 0  [0.00, 3.33] | 4 (0.12)  [0.03, 0.30] | 0.7199 |
| Vascular death | 2 (0.06)  [0.01, 0.21] | 0  [0.00, 3.33] | 2 (0.06)  [0.01, 0.21] | 0.8046 |

MACCEs, major adverse cardiac and cerebrovascular events; MI, myocardial infarction
