# Supplementary material for: Time interval of esomeprazole and dual antiplatelet therapy in patients with cardiocerebrovascular diseases
Source: Medicine (Baltimore). 2024 Mar 1;103(9):e37205. doi: 10.1097/MD.0000000000037205 (PMC10906606; doi:10.1097/MD.0000000000037205)
Supplement: Supplementary file 4 [file medi-103-e37205-s004.docx]

**Supplementary Table 3. Cox- proportional hazard regression analysis.**

## 1:1 matching PSM

|  | Unadjusted HR (95% CI) | P | Adjusted HR (95% CI)  Model 1 | P | Adjusted HR  (95% CI)  Model 2 | P |
| --- | --- | --- | --- | --- | --- | --- |
| MACCEs |  |  |  |  |  |  |
| Interval-based use | Ref |  | Ref |  | Ref |  |
| Concurrent use | 0.99 [0.06, 15.77] | 0.9921 | NA | NA | NA | NA |
| Stroke |  |  |  |  |  |  |
| Interval-based use | Ref |  | Ref |  | Ref |  |
| Concurrent use | NA | NA | NA | NA | NA | NA |
| MI |  |  |  |  |  |  |
| Interval-based use | Ref |  | Ref |  | Ref |  |
| Concurrent use | NA | NA | NA | NA | NA | NA |
| Vascular death |  |  |  |  |  |  |
| Interval-based use | Ref |  | Ref |  | Ref |  |
| Concurrent use | NA | NA | NA | NA | NA | NA |

## IPTW(Stabilized)

|  | Unadjusted HR (95% CI) | P | Adjusted HR  (95% CI)  Model 1 | P | Adjusted HR  (95% CI)  Model 2 | P |
| --- | --- | --- | --- | --- | --- | --- |
| MACCEs |  |  |  |  |  |  |
| Interval-based use | Ref |  | Ref |  | Ref |  |
| Concurrent use | 1.96 [0.26, 14.56] | 0.5113 | 1.25 [0.15, 10.41] | 0.8364 | 1.16 [0.14, 9.65] | 0.8882 |
| Stroke |  |  |  |  |  |  |
| Interval-based use | Ref |  | Ref |  | Ref |  |
| Concurrent use | NA | NA | NA | NA | NA | NA |
| MI |  |  |  |  |  |  |
| Interval-based use | Ref |  | Ref |  | Ref |  |
| Concurrent use | NA | NA | NA | NA | NA | NA |
| Vascular death |  |  |  |  |  |  |
| Interval-based use | Ref |  | Ref |  | Ref |  |
| Concurrent use | NA | NA | NA | NA | NA | NA |

# Covariates of adjusted Cox-proportional hazard regression model

Model 1: age, sex, BMI, index event (stroke, coronary artery disease), center

Model 2: age, sex, BMI, hx of stroke, history of coronary artery disease, index event (stroke, coronary artery disease), center
