# Supplementary material for: Time interval of esomeprazole and dual antiplatelet therapy in patients with cardiocerebrovascular diseases
Source: Medicine (Baltimore). 2024 Mar 1;103(9):e37205. doi: 10.1097/MD.0000000000037205 (PMC10906606; doi:10.1097/MD.0000000000037205)
Supplement: Supplementary file 5 [file medi-103-e37205-s005.docx]

Supplementary Figure 1. K-M Plot of primary endpoint variable (MACCEs) using stabilized IPTW method.

## IPTW(Stabilized)

**
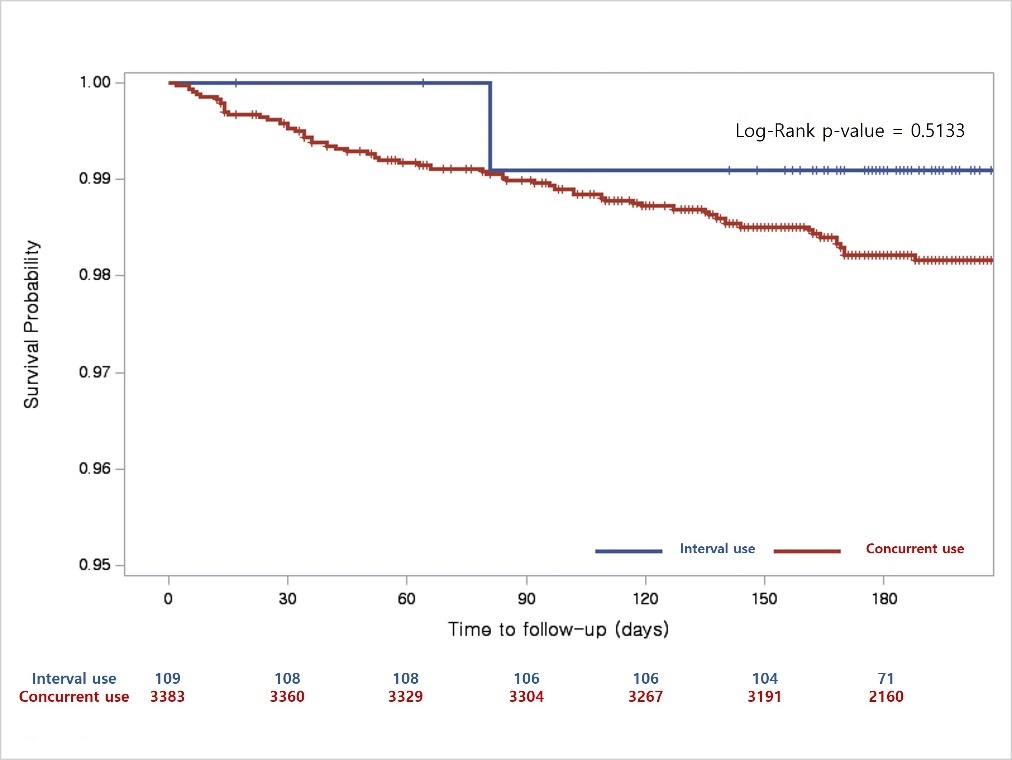
**

MACCEs = major adverse cardiocerebrovascular events; IPTW = inverse probability treatment weight
